# Defining and modeling ‘cure’ in early-stage melanoma: perspectives from a systematic literature review

**DOI:** 10.3389/fonc.2026.1824831

**Published:** 2026-08-14

**Authors:** Elizabeth Wehler, Jennifer Sander, Sophie Boukouvalas, Christopher M. Black

**Affiliations:** 1 Health Economic and Decision Sciences, Merck & Co., Inc, West Point, PA, United States; 2 Avalere Health, New York, NY, United States; 3 Avalere Health, Athens, Greece; 4 Value and Implementation, Outcomes Research, Merck & Co., Inc, Rahway, NJ, United States

**Keywords:** SLR, adjuvant therapy, cure definition, cure models, early-stage melanoma, systematic literature review

## Abstract

**Background:**

Defining “cure” in oncology remains conceptually and methodologically challenging, particularly in early-stage melanoma treated with curative intent. Recent advances in adjuvant and neoadjuvant immunotherapy have improved relapse-free and event-free survival, raising questions about how and when recurrence-free patients can be considered cured. This review aimed to clarify how “cure” is defined, identified, and modeled in early-stage melanoma and to describe the clinical and methodological evidence supporting cure assumptions.

**Methods:**

A systematic literature review (SLR) was conducted in MEDLINE and Embase from January 2014 to July 2024 using a predefined PICOTS framework. Eligible studies described definitions of “cure,” criteria for identifying cure, or quantitative modeling of long-term outcomes in solid tumors, with a focus on melanoma. Data were screened and extracted using a hybrid AI-assisted process consistent with PRISMA standards.

**Results:**

Twenty-six publications were included and grouped into analytical, data driven review, and expert-opinion categories. Most defined cure statistically, based on survival plateaus or estimated cure fractions (CFs), which ranged widely from 28% in cases with nodal category 3, to 99% for cases with localized disease according to summary stage at diagnosis. Cure estimates varied by disease stage, age, gender, and tumor site. Few studies compared cure across treatment modalities, reflecting ongoing methodological challenges with overall survival data maturity and confounding, as well as uncertainty about progression-free survival (PFS) or relapse free survival (RFS) as reliable surrogates for cure.

**Conclusions:**

Cure in early-stage melanoma remains variably defined across clinical and statistical contexts. Standardized terminology, biologically grounded definitions, and mature long-term datasets are needed to support consistent and credible modeling of curative outcomes.

## 1 Introduction

Disease recurrence remains a critical challenge for patients with early-stage melanoma who have undergone standard-of-care (SoC) treatments with curative intent, including surgery, and despite advances in treatment, many still face the risk of recurrence years after initial treatment (1, 2). Recent advances in adjuvant and neoadjuvant immunotherapy (IO) have improved event-free survival (EFS) and relapse-free survival (RFS) in high-risk stage II and resectable stage III melanoma (3–6), raising critical questions about how and when recurrence-free patients can be considered cured.

The definition of “cure” in oncology is inherently complex and varies widely depending on factors such as cancer type, stage at diagnosis, treatment administered, and timing of the cure assumption (7–11). The definition also varies among healthcare practitioners (HCPs), including oncologists and nurses, and is often context-dependent, shifting based on the audience and timing of discussions (9, 10, 12). Clinicians typically define cure as the complete absence of disease in an individual, while statisticians model cure probabilistically, estimating the proportion of patients whose mortality returns to that of the general population within a defined time frame (10).

In early-stage cancers, defining cure is particularly challenging because long-term, patient-relevant outcomes, such as RFS and EFS, may not reliably predict overall survival (OS), with cure not only as a matter of prolonged survival but also living free from disease recurrence (13–15). Although intermediate endpoints have been validated as surrogates for OS in several tumor types, their ability to reflect biological cure remains variable in associative/predictive value (16–20). Additionally these endpoints present challenges related primarily due to limited follow-up duration and censoring of data, which can hinder accurate estimation of cure metrics such as time-to-cure and cure fraction (11, 21).

Multiple factors add further complexity to the definition of cure, including variations in tumor type, prognostic and predictive biomarkers, and patient-specific characteristics such as age, disease stage, nodal status, and performance status (10, 22–32). In solid tumors, factors such as residual cancer burden, programmed death-ligand 1 (PD-L1) expression, molecular subtypes such as BRAF mutations, circulating tumor DNA (ctDNA), and molecular measures of minimal residual disease may influence cure potential, but their roles remain poorly understood (24, 32). These conventional and emerging prognostic factors underscore the need for cancer-specific approaches to defining and modeling cure. Understanding the interplay of these variables is essential for interpreting, supporting, and critiquing cure assumptions across different stakeholders, including clinicians, researchers, and payers (10). Addressing these complexities through robust, cancer-specific frameworks will be essential to refine the definition of cure and to strengthen its application in clinical research and survival modeling.

The concept of curative therapies in the adjuvant and neoadjuvant settings is relatively recent, and no universally accepted definition of cure exists across cancer types (10, 33). While neoadjuvant therapy aims to reduce tumor burden and enhance surgical outcomes, adjuvant therapy targets residual disease to lower recurrence risk (34, 35). Together, these strategies expand opportunities for durable remission in patients with localized disease.

Perceptions of cure can vary significantly across heterogeneous patient populations within a single cancer type, influenced by factors such as disease stage, tumor biology, and individual response to therapy (26, 27, 30, 36). This variability underscores the need for a deeper understanding of when and how adjuvant and neoadjuvant therapies may contribute to achieving cure.

Over the past decade, the therapeutic landscape of melanoma has been transformed by an improved understanding of its molecular drivers. Large-scale genomic efforts such as The Cancer Genome Atlas (TCGA) have delineated biologically distinct subtypes defined by mutations in BRAF, NRAS, NF1, and KIT, providing the foundation for targeted treatment approaches (37). Among these, combined BRAF and MEK blockade has demonstrated durable relapse-free and OS benefits in the adjuvant setting, as evidenced by long-term results from the COMBI-AD trial (38, 39). Similarly, immune checkpoint inhibitors, such as nivolumab and pembrolizumab, both anti–programmed cell death protein 1 (anti–PD-1) agents, have significantly enhanced outcomes for patients with high-risk melanoma, as evidenced by pivotal trials like CheckMate 238 (nivolumab vs. ipilimumab) and KEYNOTE-054 (pembrolizumab vs. placebo) (40, 41). Early-phase studies in the neoadjuvant context further suggest that upfront molecularly guided therapy can eradicate micrometastatic disease and achieve high pathological response rates (42). While similar precision strategies for NRAS, KIT, and NTRK alterations are emerging, their integration into curative-intent paradigms remains nascent (43–45).

These molecular insights have not only transformed melanoma management but also redefined the boundaries of what constitutes potential cure. Collectively, these advances have reframed melanoma from an invariably recurrent malignancy to one in which long-term remission, and possibly cure, appears attainable for a growing subset of patients including those treated with former standard of care (SoC) as well as newer approaches, regardless of mutation status, though rates of cure may vary depending on treatment modality.

The objective of this systematic literature review (SLR) was to better understand how “cure” is defined for patients with early-stage solid tumors treated with definitive, curative-intent therapy including adjuvant or neoadjuvant treatment. In this manuscript, melanoma is used in a test case because immunotherapy creates long tails and delayed effects, stressing cure models. Specifically, this review sought to characterize the clinical criteria used to identify cure and examine how cure is modeled in economic evaluations. By addressing these questions, this review seeks to clarify how cure is conceptualized and modeled in early-stage melanoma, providing a foundation for consistent, evidence-based application in clinical and economic decision-making.

## 2 Methods

A broad SLR covering seven tumor types^1^ was conducted for the period January 2014 to July 2024 to identify publications addressing the clinical definition of “cure” and related assumptions, such as cure time point and proportion of patients considered to be cured in the context of adjuvant and neoadjuvant therapy. While our review encompassed multiple tumor types, we specifically focused on resectable melanoma, which has seen rapid advances in treatment and survival outcomes. This makes melanoma an ideal setting to examine evolving definitions of “cure” and related assumptions. For this SLR, we included studies providing data specific to resectable melanoma, acknowledging that neoadjuvant therapy has not yet received regulatory approval in this indication (see Appendix A for a list of articles included for other tumor types) (46). A 10-year window was selected to encompass the period during which the concept of “cure” began to emerge in solid tumor research, and to align with the timing of most adjuvant and neoadjuvant therapy approvals. The literature search was conducted on July 10, 2024, and a 10-year time horizon was maintained to exclude potentially outdated definitions and modeling approaches of cure. This timeframe also coincides with the first HTA appraisal to reference “cure” in early-stage melanoma (NICE TA544) (dabrafenib with trametinib for adjuvant treatment of resected BRAF V600 mutation-positive melanoma, 2018) (47).

The purpose of this SLR was qualitative and exploratory rather than quantitative. It aimed to examine how “cure” is defined and conceptualized across solid tumors, how terminology and frameworks support communication among clinical and health-economic stakeholders, and how different methodological approaches influence cure metrics. Within the melanoma-focused subset, the review additionally sought to identify published estimates of cure metrics, (e.g. cure fractions, time-to-cure, conditional and relative survival) and the modeling approaches used (e.g., mixture cure models [MCMs] and flexible parametric cure models [FPMs]) relevant to patients with early-stage disease receiving definitive treatment with adjuvant or neoadjuvant therapy.

Searches were conducted according to the Cochrane Handbook for Systematic Reviews of Interventions, and reported following the PRISMA (Preferred Reporting Items for Systematic Reviews and Meta-analyses) guidelines (48). The search strategy was structured using the PICOTS framework (Population, Intervention, Comparator, Outcomes, Time frame, and Study design; Appendix B) Electronic searches were performed in MEDLINE In Process/PubMed via Pubmed.gov, and Embase via Embase.com, with results deduplicated across databases. Full search strategies are presented in Appendix C.

To supplement database findings, electronic searches of major conference proceedings (ASCO, ESMO, and ISPOR annual European and international meetings, 2020–2024) were conducted using PICOTS-based keywords. Abstracts were included if they contained key words related to cancer (i.e., cancer, oncology, malignancy), specified the type of cancer and disease stage (i.e., neoadjuvant, adjuvant), and referenced cure-related terms (i.e., cure, curative). This 4-year limit was applied under the assumption that earlier high-quality abstracts would have since been published as full-text articles.

SLR inclusion criteria were necessarily broad to ensure no articles were missed given that it was possible that articles mention “cure” in the discussion section rather than in the title or abstract. Studies from all geographies were included, and articles published before January 2014 were excluded. All excluded articles were coded with a reason for exclusion based on the PICOTS framework (see Appendix B). Clinical trials identified through the search strategy were not screened at the title/abstract stage. To maintain transparency and prevent inadvertent exclusion of relevant literature, pre-defined non-relevant categories such as pediatric-only populations, metastatic or advanced-stage disease, and non-clinical designs (e.g., case reports, *in vitro*, or *in vivo* studies), were tagged to enable consistent exclusion during both screening levels. Pediatric-only populations were excluded to avoid introducing heterogeneity into the study, as this population differs from adults in clinical characteristics, treatment approaches, and outcomes. Although articles with a primary focus on surgery were excluded at title/abstract screening, a small number of publications that included surgical outcomes incidentally but contained relevant findings were retained.

The identified publications from the literature databases were screened at two levels: Level 1: title/abstract, Level 2: full text. The screening was based on the set of pre-defined inclusion criteria and exclusion criteria. Articles had to meet all the inclusion criteria to be included; articles were excluded if they met at least one of the exclusion criteria. Searches were managed, deduplicated and screened using the bibliographic management software platform Nested Knowledge.

Title and abstract screening (Level 1) was performed using an AI-assisted hybrid approach (human reviewer plus AI) within a web-based systematic review platform. AI-assisted screening was used to increase efficiency, however, due to current limitations in AI accuracy, continuous human oversight was essential to maintain quality control and uphold rigorous standards (49). Two reviewers first screened a training sample of 50 articles to calibrate inclusion and exclusion decisions, after which the AI model was iteratively refined and validated (target predictive accuracy AUC ≥ 0.98). The AI then prioritized remaining records for human review, with continuous oversight to ensure quality control and adherence to predefined criteria. Full-text screening (Level 2) was then performed independently by two reviewers, with discrepancies resolved by consensus or third-party adjudication. Reasons for exclusion were documented at each stage (see Appendix D for detailed model parameters, inclusion thresholds, and quality assurance procedures).

**Figure 1:**
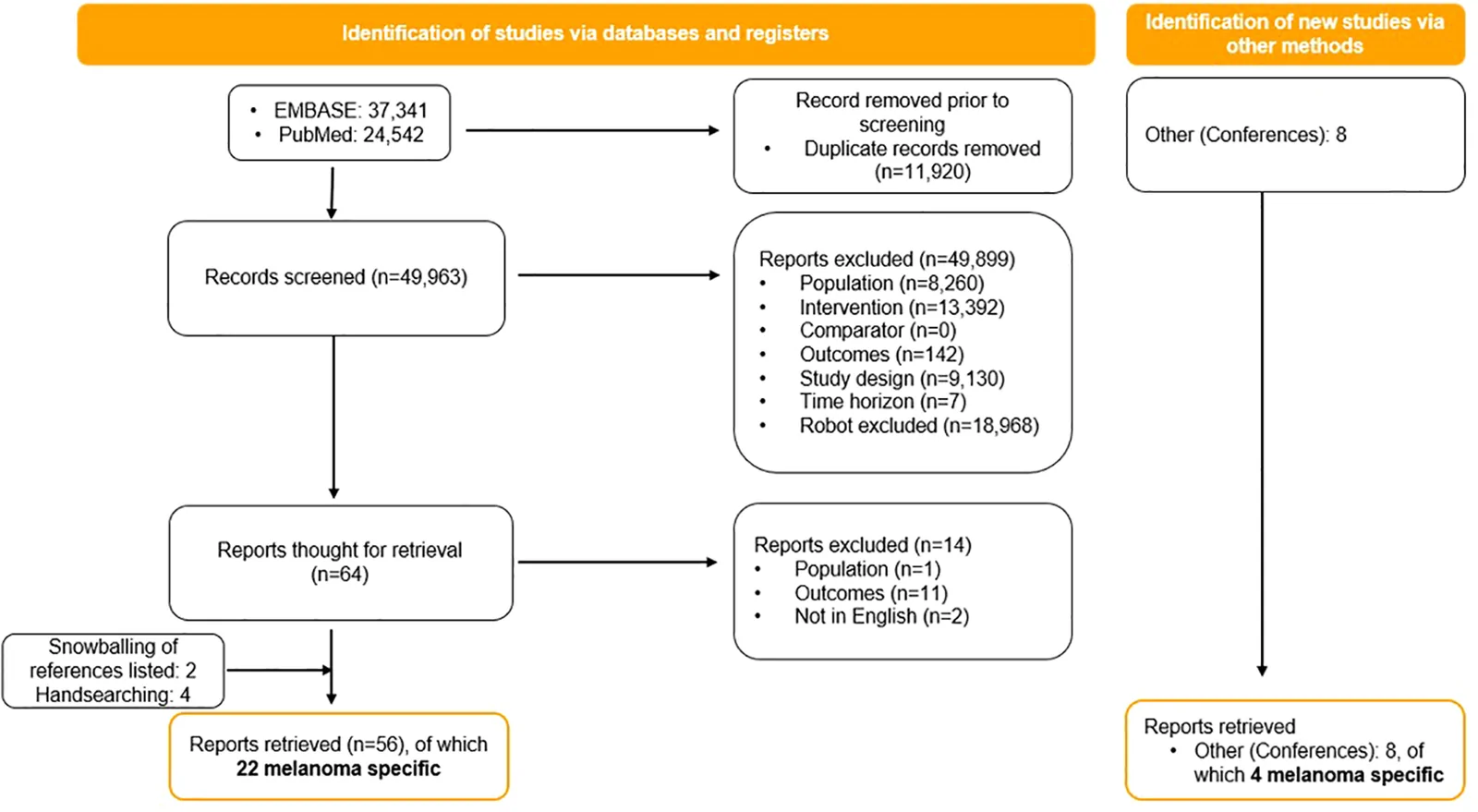
PRISMA chart.

Bibliographies of any identified SLRs and meta-analyses published within the past 5 years were reviewed to identify additional relevant studies using the ‘Bibliomine’ functionality within the review platform. The results of the screening process were reported in a PRISMA flow diagram which describes the study selection process, reasons for exclusion per level screening, and the number of publications selected for inclusion (**Figure 1**).

All eligible studies proceeded to data extraction. Data extraction was performed by trained reviewers using a tailored data extraction table. When multiple publications reported findings from the same dataset, information was integrated to ensure completeness. All data extracted were quality-checked by a senior reviewer. Following data extraction, thematic analysis was conducted to identify and synthesize key patterns and themes across the included studies.

## 3 Results

Following deduplication, 49,963 articles were screened. Fifty full texts passed screening criteria, and then six more were identified through snowballing and hand-searching; these additional 6 were not originally captured due to MeSH coding. Twenty-two were melanoma-specific. In addition, eight conference abstracts were retrieved, four of which were melanoma-specific, yielding a total of 64 pieces of literature, including 26 melanoma records for analysis. The most frequent reasons for exclusion were absence of definitive curative-intent treatment, inclusion of advanced or metastatic populations, and ineligible study design as defined by the PICOTS framework. Full search strategies are presented in Appendix C.

### 3.1 Cure definition across oncology

A total of 20/64 (31%) publications, including both melanoma and non-melanoma, mentioned “cure” for patients with early-stage cancer; of these, 12/20 (60%) were specific to the adjuvant or neoadjuvant setting. However, most publications mentioning ‘cure’ did not focus solely on early-stage cancers. Due to this limited information, additional insights were obtained from 44 studies including a broader population (i.e. some patients with advanced-disease). In these studies, relevant information pertaining principally to early-stage cancer was extracted wherever possible, although in some cases the information may have applied to both early-stage and advanced disease populations.

### 3.2 Cure lexicon by perspective

Across studies, three core interpretations of “cure” were identified (8, 9, 11):

- Lack of disease progression, defined as patients remaining recurrence-free for the duration of long follow-up (e.g. ≥ 5 years) (11), or achieving “persistent clinical remission” (8).
- Survival (statistical or population cure), defined as: mortality returning to that of the general population (8), or a plateau in the relative survival curve (9).
- Eradication of cancerous cells (medical cure), defined as elimination of all cancer cells, with no recurrence until death from other causes (9).

Within the medical cure concept, three sub-themes were also noted:

- Use in practice: claiming eradication is seldom measurable because undetectable malignant cells may remain post-therapy (50).
- Morbidity considerations: equating cure with the elimination of both cancer mortality and the broader morbidity burden (51).
- Post-surgical cure: defining cure pragmatically as remaining metastasis-free after surgery with curative intent (52).

Within the articles, cancers and treatments were commonly grouped into three outcome categories:

- Palliative: disease controlled but not eradicated, with ongoing or periodic treatment (8).
- Curative: transformative outcomes for most patients, (50) often defined by > 5 years survival with no excess mortality (8) characterized by a plateau in relative survival curves (53–56), or by achieving survival rates comparable to those of the general population (57).
- Mixed: substantial outcomes achieved only in a minority, with limited benefit for the remainder of patients (27).

### 3.3 Methodological approaches

The included publications were grouped into four categories based on methodological approach:

- Analytical approach for cure models: publications that explored or described methodological frameworks, techniques, or structural variations in existing cure model approaches,
- Data analyses: publications that applied established cure modeling methods, including cost-effectiveness analyses, to existing datasets, reporting or discussing derived cure-related estimates (e.g. cure fraction [CF], time-to-cure [TTC]) to illustrate these methods,
- Review: publications that approached cure from an academic or conceptual perspective, including literature reviews,
- Expert opinion and/or consensus: articles that used primary qualitative research to capture professional perspectives on cure.

Cure metrics were highly dependent on the analytical framework and endpoints applied. Traditional endpoints such as OS, PFS, and RFS remain dominant despite their limitations for assessing curative potential (9). Authors have highlighted the need for new or composite metrics better suited to therapies with curative intent (9, 58).

CF estimates vary according to both endpoint choice and modeling framework. Three principal frameworks were identified: disease-specific, all-cause, and relative survival (RS) (9, 11, 58). In disease-specific frameworks, models are fitted to cause-specific survival functions and later combined with general-population mortality. All-cause frameworks model total survival but require decisions on how to embed background mortality. Common approaches for embedding background mortality include using standardized mortality ratios (SMRs), lifetable adjustments, and relative survival models. Double counting can occur if background mortality is added to disease-specific hazards without properly accounting for overlap since deaths attributed to the disease are already included in all-cause population mortality rates. This can bias survival estimates by counting the same death as both disease-related and as a background (non-disease) event, thereby inflating overall hazard rates and underestimating survival or cure fractions. RS frameworks incorporate population mortality directly and avoid reliance on cause-of-death data; they often yield higher CF estimates because deaths from other causes are excluded (58).

MCMs and non-mixture cure models (NMCs) estimate cure in different ways. In MCMs selecting a suitable distribution for the uncured sub-population is key, whereas in NMCs the focus is on modeling the hazard function up to the cure timepoint (58). Analytic challenges include limited follow-up and censored data (31).

The use of traditional endpoints in the most common way results are presented in literature. As far back as 2015, a review by Johnson and colleagues found that endpoints used in the traditional context of chemotherapy, radiation, and surgery were frequently in use in both clinical studies and health technology assessments (9). They spoke about the need for new metrics to describe the medical value of “new” therapies with curative potential for solid tumors, suggesting appropriate endpoints (e.g. mean OS, cure fraction, and OS rate at landmark time points) to measure long-term survival in clinical trials and practice (9). OS is the most objective endpoint for cure-related inference; the limitation is data maturity, competing risks, and post-progression therapy confounding, not that OS is intrinsically unfit. PFS/RFS are often the earliest indicators of durable benefit; the limitation is surrogacy, uncertainty and late relapse risk. If endpoints used to define and determine estimates of cure cannot adequately capture the curative potential outcomes of these therapies, alternative metrics should be considered (9).

### 3.4 Melanoma sub-set results

Of the 26 melanoma-related publications, 13 focused exclusively on melanoma and 13 analyzed melanoma alongside other tumors. Eleven explored cure-modeling techniques (analytical approaches), and eleven applied data analyses within cure-model frameworks, including two publications employing cost-effectiveness modeling. The remaining four were HTA/literature reviews (n = 3) or expert/qualitative perspectives (n = 1).

Five of 13 melanoma-only publications (38%) explicitly defined “cure” and provided melanoma-specific cure metrics, 5/13 (38%) defined “cure” without reporting metrics, and 3/13 (24%) assessed cure statistically without a written definition, rather assessed cure based on statistical modeling approaches, one of which also reported CF (**Table 1**). For the 13 mixed tumor studies, 7/13 (54%) reported cure metrics.

**Table 1:** Cure lexicon included in studies that focus solely on melanoma.

Study	Type of article	Cure lexicon category	Cure definition
Andersson et al., 2014 (57)	Data analyses (cure model)	Survival/statistical	Cure proportion: *“proportion of patients that do not experience excess mortality due to the disease”*
Silversmit et al., 2017 (55)	Data analyses (cure model)		Cure: “*Cumulative relative survival curves for many cancers reach a plateau several years after diagnosis, indicating that the cancer survivor group has reached “statistical” cure”*
Harris et al., 2022 (62)	Data analyses (cure model)		*This abstract did not explicitly provide a specific definition of 'cure,' but instead evaluates cure in terms of patients remaining recurrence-free, as estimated MCMs*
Ishak et al., 2018 (53)	Analytic approaches (cure model)		Cure proportion*: “Flattening of curve suggests risk of cancer related deaths has become negligible, proportion cured aligned with observed plateau”*
Nicolaie et al., 2019 (54)	Analytic approaches (cure model)		CF: *“proportion of the population for which none of the competing events can occur”* Cure: *“patient not experiencing death due to the disease-related cause; therefore, the status of cure is observed only for individuals subjected to death not due to the cause of interest.”* *The presence of a sub-population that survived event-free was indicated by a plateau in the survival curve*
Balakrishnan et al., 2020 (23)	Analytic approaches (cure model)		*This publication did not explicitly provide a specific definition of 'cure,' but instead described the use of a statistical cure model to estimate cure rates and the probability of being cured over time, conditional on survival and nodal category.*
Gomez et al., 2021 (25)	Analytic approaches (cure model)		*This publication did not explicitly provide a specific definition of 'cure,' but instead evaluates cure in terms of overall survival as estimated by an FPM.*
Webb et al., 2022 (30)	Analytic approaches (cure model)		TTC:*“patients who are cured and will never experience the event of interest”*
Zhang et al., 2023 (64)	Analytic approaches (cure model)		*Cure: “the per-cycle risks of transitions from the recurrence-free state (as estimated under the scenario with no cure assumption) were reduced by 95% for patients who achieve RFS ≥ 10 years. This percentage reduction in recurrence risk was assumed to linearly increase from 0% at 7 years to 95% by 10 years onward.” ”*
Balakrishnan et al., 2017 (59)	Analytic approaches (cure model)	Lack of disease progression	*Cured patients: “Individuals completely cured from a certain disease and will not face any recurrence usually called cured or non-susceptible or long-term survivors or immunes”*
Calsavara et al., 2020 (60)	Analytic approaches (cure model)		*“Individuals not susceptible to the event of interest, even if, accompanied by a sufficiently large time, which is so-called immune”*
Su et al., 2022 (63)	Analytic approaches (cure model)		Cure: “*significantly prolonged interval of relapse-free”* CF: *“proportion of subjects cured of disease”*
Brandao et al., 2023 (61)	Analytic approaches (cure model)	Survival/statistical Eradication	CF: *“proportion of patients responds favorably to a treatment, thus improving overall survival”*

CF, cure fraction; FPM, flexible parametric cure model; MCM, mixture cure model; TTC, time to cure.

Two melanoma-only studies (15%) equated cure with remaining recurrence-free, avoiding the terms “cure” or “cured” and instead emphasizing absence of clinical events (59, 60). Both publications used the term “immune” in this context. Among these two publications, Balakrishnan et al., also introduced the concept of non-susceptibility, describing cure individuals as those who are no longer at risk for future events such as recurrence, sometimes referred to as immunes or long-term survivors. Eleven (85%) defined cure statistically, describing population-level plateaus in survival (23, 25, 30, 53–55, 57, 61–64); three of these relied solely on statistical modeling without a written definition (23, 25, 62). One study (8%) discussed medical/eradication cure, explicitly (61).^2^

Cure definitions across melanoma studies consistently emphasized a negligible risk of disease-related mortality, often identified by a plateau in survival curves. Most publications (10/13) adopted a statistical population-level perspective, whereas clinician and patient perspectives, typically expressed as “long-term survival”, were less represented. Additionally, one study described cured patients as those entering a recurrence-free state specifically after complete resection of stage IIB–IIC melanoma, highlighting a treatment-specific approach to defining cure status (64).

CF terminology varied, e.g. “the proportion of the population for whom none of the competing events can occur” (54), “the proportion of patients cured of disease” (63), “the proportion of patients that respond favorably to a treatment, thus improving OS” (61), or as “the probability of cure among incident cases” (65). One study used Italian registry data to compare net survival, life expectancy of fatal cases, CF, TTC, cure prevalence, and conditional net survival (65).^3^

Three studies described cure in terms of a plateau in survival, indicating that the risk of cancer-related death becomes negligible after a certain period following diagnosis or treatment (53–55). A flattening or plateau in survival curves signaled that a sub-population had achieved statistical cure, aligning with observed long-term survivors (54, 56).

#### 3.4.1 Melanoma cure metrics

Across melanoma publications, CF estimates (**Table 2**), ranged widely from 28% in cases with nodal category 3 (23), to 99% (56) for cases with localized disease according to summary stage at diagnosis. One study reported that its MCM did not converge for melanoma, preventing CF estimation (66).

**Table 2:** Cure metrics specific to melanoma.

Authors	Methodology	Data source (geography)	Variable	Cure findings	
				TTC (years)	Fraction/proportion
Andersson et al., 2014 (57)	Data analyses (cure model)	Two regional quality registers in the Uppsala–Orebro and Stockholm–Gotland health care regions in Central Sweden (diagnosis of CMM; timeframe: 1996-2005, followed up until death or 31 December 2010) (Sweden)	Stage classified into clinical stage IA, IB, II or III–IV according to the 2002 American Joint Committee on Cancer (AJCC) melanoma classification system	Not reported	• Stage IA: 98% • Stage IB: 88% • Stage II: 71% • Stage III-IV: 48%
Hubbel et al., 2024 (67)	Data analyses (cure model)	SEER database on 17 geographic regions of the US (primary cancer site using ICD-O-3; timeframe: 2006-2015) (USA)	Stage according to the 6th edition of the AJCC staging manual	Not reported	• Stage I: 95% • Stage II: 83% • Stage III: 67%
Kou et al., 2020 (27)	Data analyses (cure model)	Australian Cancer Database by the Australian Institute of Health and Welfare (AIHW), (ICD10 code C43 – malignant melanoma of the skin; timeframe: 1982-2014) (Australia)	Sex	Not reported	• 83.2% (M); 90.3% (W)
Dal Maso et al., 2014 (68)	Data analyses (cure model)	Italian cancer registries (ICD 10 code C43 – malignant melanoma of the skin; timeframe: 1985-2002, followed up until 2007) (Italy)	Sex and age at diagnosis	Not reported	• 15–44 yrs: 77% (M), 85% (W) • 45–54 yrs: 67% (M), 78% (W) • 55–64 yrs: 61% (M), 73% (W) • 65–74 yrs: 54% (M), 68% (W)
Romain et al., 2019 (70)	Data analyses (cure model)	FRANCIM (the French network of cancer registries) (ICD-O-3; timeframe: 1995-1999) (France)	Sex and age at diagnosis	• 15–44 yrs: 5.1 (M), 3.8 (W) • 45–54 yrs: 5.2 (M), 5.1 (W) • 55–64 yrs: 4.9 (M), 6.4 (W) • 65–74 yrs: 5.9 (M), 8.8 (W)	• 15–44 yrs: 84% (M), 92% (W) • 45–54 yrs: 83% (M), 90% (W) • 55–64 yrs: 85% (M), 87% (W) • 65–74 yrs: 79% (M), 80% (W)
Dal Maso et al., 2022 (8)	Expert opinion	Evidence summaries (Global)	Sex and study country	• Europe: 6 (M), 2 (W) • Italy: 6 (M), 6 (W) • France: 5 (M), 5 (W) • Europe (2): 7 (M), 2 (W) • USA: 2 (M), 2 (W) • Australia: 3 (M), 3 (W)	• Europe: 76% (M), 86% (W) • Italy: 75% (M), 83% (W) • France: 85% (M), 90% (W) • Belgium: 75% (M), 84% (W) • Australia: 83% (M), 90% (W)
Kou et al., 2020 (27)	Data analyses (cure model)	Australian Cancer Database by the Australian Institute of Health and Welfare (AIHW), (ICD10 code C43 – malignant melanoma of the skin; timeframe: 1982-2014) (Australia)	Age at diagnosis	Not reported	• 15–49 years: 92.1% • 50–69 years: 88% • 70–89 years: 83.6%
Eloranta et al., 2014 (69)	Analytic approaches (cure model)	Swedish Cancer Registry (ICD7 code 190x – malignant melanoma; timeframe: 1973-2007, follow up until 31 Dec 2012) (Sweden)	Age at diagnosis	Not reported	• 50: 85% • 60: 83% • 70: 77% • 80: 70%
Hubbel et al., 2024 (67)	Data analyses (cure model)	SEER database on 17 geographic regions of the US (primary cancer site using ICD-O-3; timeframe: 2006-2015) (USA)	Age at diagnosis	Not reported	• 40–54 yrs: 98% (Stage I), 85% (Stage II), 70% (Stage III) • 55–64 yrs: 96% (Stage I), 83% (Stage II), 66% (Stage III) • 65–74 yrs: 93% (Stage I), 81% (Stage II), 63% (Stage III) • 75–84 yrs: 89% (Stage I), 70% (Stage II), 49% (Stage III)
Xia et al., 2022 (56)	Data analyses (cure model)	SEER database (ICD-O-3; timeframe: 1975-2018) (USA)	Summary stages according to spread of disease at diagnosis	• Localized: 0 • Regional: 6.1 • Distant: 4.5	• Localized: 99.2% • Regional: 65.9% • Distant: 30%
Balakrishnan et al., 2017 (59)	Analytic approaches (cure model)	Dataset from Ibrahim et al., 2005 (the data are part of a study on cutaneous melanoma for the evaluation of postoperative treatment performance with a high dose of interferon alpha-2b as a drug to prevent recurrence; timeframe: 1991-1995, follow up until 1998)) (Geography not reported)	Nodule category	Not reported	• 1: 65% • 2: 54% • 3: 43% • 4: 32%
Balakrishnan et al., 2020 (23)	Analytic approaches (cure model)	Phase III clinical trial conducted by the ECOG, E1690. (Geography not reported)	Nodal category	Not reported	• 0: 62% • 1: 50% • 2: 39% • 3: 28%
Silversmit et al., 2017 (55)	Data analyses (cure model)	Belgian Cancer Registry (skin melanoma (ICD-10 C43, cases with unknown localization (ICD-O-3 C80.9) were excluded); timeframe: 1999-2011) Belgium	Sex and age	Not reported	• Overall: 80.8% • Male: 75.3% • Female: 84.2% • 15–34 years: 89.0% • 35–64 years: 82.3% • 65+ years: 73.9%
Nicolaie et al., 2019 (54)	Analytic approaches (cure model)	Andersen et al. (1993): Melanoma cancer dataset from University Hospital of Odense in Denmark; timeframe: 1962-1977 (Denmark)	Not applicable	• 10 years (plateau in survival curves)	Not reported
Wang et al., 2020 (72)	Analytic approaches (cure model)	Andersen et al. (1993): Melanoma cancer dataset from University Hospital of Odense in Denmark; timeframe: 1962-1977 (Denmark)	Not applicable	• 8 years (plateau at the tail after nearly 3000 days)	Not reported
Zhang et al., 2023 (64)	Data analyses (cure model)	KEYNOTE-716, KEYNOTE-006 and network meta-analysis data; stage IIB or IIC melanoma (USA)	Not reported	• 7–10 years • (linearly increase from 0% at 7 years to 95% by 10 years onward)	Not reported

Population heterogeneity substantially influenced the estimates of cure metrics (54). Factors affecting CF and TTC included:

- Disease stage: CF typically declined with increasing stage of disease (8, 25, 27, 57, 67). One publication (Andersson 2014) identified stage as the strongest determinant of cure (57). Reduction ranging from 28% to 50% between Stage I and Stage III/IV (57, 67).
- Tumor site: location of tumor influenced CF, with head/neck melanomas showing lower cure rates than extremity tumors (30, 54, 57).
- Age: most studies found higher CF in younger patients (25, 27, 54, 55, 57, 63, 67–70); although Webb 2022 did not (30). Differences were sometimes attributed to competing-risk effects or treatment intensity (69). One study considered that the impact of age on CF was less than the impact of other factors like disease stage and site (54), and estimated TTC was longer in older age (65–74 years) patients compared to younger age group (15–44 years) for both men and women (70).
- Gender: females generally had higher CFs and shorter TTCs (2 vs 7 years) than males (8, 25, 27, 54, 55, 68-70). One study (Romain et al.) reported higher TTC in females than males, except for the oldest age group (70).
- Other factors: increasing tumor thickness reduced CF (30, 59); prior surgery improved survival, whereas education level, prior radiotherapy, or prior chemotherapy had minimal effect (25). A further study found that prior surgery did impact cure rates in later stage melanoma but there was little impact in early-stage disease (67).

Real-world data indicate that adjuvant immunotherapy following surgery may yield poorer survival outcomes than clinical trial populations, but there is confounding by indication, typical shift in trial vs. RWD patient case mix, follow-up is different, and heterogenous practice (71). One review summarized CFs of 75 – 85% for men and 83 – 90% for women, with TTC ranging 2–7 years (65).^4^ A conference abstract comparing an MCM with a standard parametric approach for adjuvant nivolumab found that cure modeling better captured long-term survival tails (62). The results indicated that cure modeling can materially change extrapolated survival and ICER estimates; direction depends on assumptions (62).

These factors all highlight the need to take account of population heterogeneity when interpreting cure metrics, and discussions with patients need to be personalized to account for the impact of these differences (8).

#### 3.4.2 Model approaches used to estimate melanoma cure metrics

Five studies used relative survival (RS) to summarize outcomes or model cure (27, 55, 57, 66, 69). RS was typically expressed as the proportion of patients alive assuming cancer was the only cause of death, with population cure inferred where RS curves plateaued (8, 27, 66). CRS was discussed in two studies, which defined TTC as time to reach 5-year relative survival >95% (8, 68) (**Table 3**). Conditional net survival (CNS) was also reported as a dynamic measure of prognosis over time in a population-based, observational methodological study using real-world cancer registry data. (65).

**Table 3:** Model approaches used to generate cure outcomes.

Authors	Methodology	Variables	Model approach/type
Andersson, et al., 2014 (57)	Data analyses (cure model)	RS	FPM
Romain, et al., 2019 (70)	Data analyses (cure model)	NR	FPM
Balakrishnan et al., 2017 (59)	Analytic approaches (cure model)	NR	MCM estimated via EM algorithm
Balakrishnan et al., 2020 (23)	Analytic approaches (cure model)	NR	Generalized mixture (binary) cure rate model
Edlinger, et al., 2014 (66)	Data analyses (cure model)	RS	Mixture cure proportion models
Nicolaie, et al., 2019 (64)	Analytic approaches (cure model)	NR	MCM using logistic regression for incidence and Cox proportional hazards model for latency, with piecewise constant hazards.
Zhang et al., 2023 (64)	Data analyses (cure model)	NR	Cost-effectiveness Markov model
Wang et al., 2020 (72)	Analytic approaches (cure model)	NR	Semi-parametric model based on the sieve ML estimation under the MCM
Dal Maso et al., 2022 (8)	Expert opinion	CRS	Parametric mixture or flexible models
Eloranta et al. (69)	Analytic approaches (cure model)	RS	FPM used in combination with competing-risks theory
Kou et al. (27)	Data analyses (cure model)	RS	FPMs
Hubbell et al. (67)	Data analyses (cure model)	NR	MCM
Dal Maso 2014 (68)	Data analyses (cure model)	CRS	MCMs
Silversmit et al. (55)	Data analyses (cure model)	RS	Non-linear parametric MCMs

CRS, conditional relative survival; EM, expectation–maximization; FPM, flexible parametric cure model; MCM, mixture cure model; ML, maximum likelihood; NR, not reported; RS, relative survival.

Among the 13 studies reporting melanoma-specific cure metrics, eight employed MCMs (8, 23, 54, 59, 66-68, 72) and five used FPMs (8, 27, 57, 69, 70) (**Table 3**). Specialized variants included semi-parametric, non-linear and generalized mixture models, designed to handle heterogeneous risk or competing-event structures. One study explicitly incorporated competing-risk theory to partition deaths due to melanoma versus other causes, improving accuracy of long-term survival estimates. A cost-effectiveness analysis using a Markov model assumed a 7-10-year period as the time to cure for patients with early-stage melanoma (64).

## 4 Discussion

The findings of this review highlight both conceptual and methodological challenges in defining and modelling “cure” across oncology, with particular complexity observed in melanoma.

### 4.1 Broader literature context

Across the oncology literature, no universally accepted definition of “cure” exists, and its interpretation varies by discipline. Most frameworks converge on three key conceptualizations: (1) lack of disease progression, (2) eradication of cancerous cells, and (3) survival beyond the expected disease-specific mortality (8, 9, 11). Perspectives also differ by stakeholder group: healthcare professionals and academics tend to emphasize disease progression and survival; HTA agencies focused mostly on ‘lack of disease progression (in the absence of mature overall survival data)’; epidemiologists focus on statistical cure defined through population survival metrics and patients often describe a ‘psychological cure,’ referring to freedom from fear of recurrence (9, 10, 12).

Cure estimates across solid tumors remain highly heterogeneous, reflecting differences in stage distribution, tumor biology, patient characteristics, and analytical methods (8, 25, 27, 30, 54, 55, 57, 59, 63, 67–70), and depends on methodological approaches used (11, 31, 58). Estimating the proportion cured after early treatment is particularly challenging due to limited follow-up and difficulty distinguishing patients who are truly cured from those yet to relapse, compounded by limited follow-up time and censoring. (11, 21, 31). While models frequently apply standard cure metrics, such as CF or TTC, there is no consensus on their operational use or thresholds (8, 10, 12, 27, 54, 65, 70, 73–75). Although statistical models have evolved to estimate cure in diverse ways, the clinical plausibility of the long-term estimates remains critical (73, 74).

Methodologically, MCMs and FPMs have expanded the statistical toolkit for quantifying long-term outcomes. However, the validity of their outputs depends heavily on data maturity. As highlighted by Latimer and colleagues, early data cuts can produce unstable or inflated CFs, underscoring the importance of sensitivity testing, clinical validation, and external benchmarking before interpreting apparent survival plateaus as evidence of cure (58). These considerations are particularly relevant in immuno-oncology, where delayed treatment effects and long-term survivors may mimic, but not confirm, statistical cure.

Beyond modelling technique, the robustness of data sources is critical. Data used to justify cure assumptions, whether trial extensions, registries, or real-world datasets, must align with local clinical practice and population characteristics. Failure to ensure this comparability risks biasing survival estimates or overestimating the proportion cured. Clear documentation of data provenance and validation is therefore an essential component of credible cure modelling.

### 4.2 Melanoma-specific discussion

In melanoma, defining and modelling cure remain especially complex. Most publications focus on population-level or statistical cure, inferred from survival plateaus, while very few explicitly define cure in biological or clinical terms. Consistent with the broader oncology literature, cure estimates in melanoma vary substantially by disease stage, tumor site, and demographic factors such as age and gender (8, 25, 27, 30, 54–57, 59, 63–70). Reported CF estimates range widely, from 28% (23) to 99% (56), and TTC values typically fall between 2 years and 10 years, depending on the modelling framework, covariate adjustments, and maturity of follow-up data (8, 56, 66, 67) (**Table 2**).

The recently published Weber et al. (2024) analysis, although outside the temporal scope of this review, provides valuable complementary insights. Using extended follow-up data from pivotal adjuvant trials, Weber and colleagues modelled long-term survivorship among patients with resected stage III–IV melanoma treated with adjuvant immunotherapies (nivolumab, ipilimumab, and placebo). Their results demonstrate that cure modelling can discern treatment-level differences in durable survival, supporting the feasibility of mixture-cure approaches when sufficiently mature data are available. The study also reinforces that the definition of “cure” must remain closely linked to the biological and temporal characteristics of each therapy and dataset (76).

No included study directly compared cure rates across different treatment modalities, highlighting a major evidence gap. This likely reflects both definitional heterogeneity and the historical lack of sufficiently mature, comparable datasets in early-stage melanoma. Future research should therefore explore treatment-specific cure estimates using harmonized modelling frameworks and long-term, pooled datasets to determine whether adjuvant or neoadjuvant interventions meaningfully shift the proportion of patients achieving durable remission. Building on these observations, several priorities for future research and standardization can be identified.

### 4.3 Future directions

The absence of consistent cure definitions across clinical, statistical, and patient perspectives underscores the need for a harmonized lexicon. Definitions should be tailored to audience and purpose, for example statistical cure for epidemiological and modelling contexts versus disease-free survival or clinical remission for clinical communication. The evolution of the treatment landscape further complicates specifying a single timepoint for cure, as advances may shift these benchmarks over time. Additional research is necessary to bridge the gap between statistical and clinical definitions of cure to help translate evolving definitions of cure into meaningful clinical recommendations that improve patient care.

In early-stage melanoma, long-term overall survival data remain immature; thus, relapse-free survival (RFS), distant metastasis-free survival (DMFS), and event-free survival (EFS) should be recognized as clinically meaningful endpoints that capture durable benefit with long follow-up validation needed. Promoting the adoption of these intermediate outcomes, as opposed to validating them solely as surrogates for OS, would facilitate earlier, evidence-based evaluation of potentially curative therapies. Advances in technology may further refine staging accuracy, thereby improving assessment of cure potential and optimizing patient stratification (77).

Further, the credibility of any cure assumption depends on the maturity, representativeness, and quality of the data underpinning it. Robust justification for data source selection, transparency in model assumptions, and incorporation of external and clinical validation are essential to strengthen confidence in cure metrics. For future submissions, a proposed minimum reporting checklist for cure modeling in HTA/oncology should be included (data maturity, external validation, sensitivity analyses, endpoint definitions, background mortality handling).

Future work should explore the concept of ‘cure’ in collaboration with key stakeholders to acknowledge and integrate the diverse interpretations held across different groups. Future economic modeling should incorporate flexible frameworks that capture heterogeneity in treatment effects and explicitly quantify uncertainty in long-term outcomes.

### 4.4 Limitations

While this review provides a structured overview of evolving cure concepts and modelling strategies, several limitations should be acknowledged. This review synthesized publications from 2014 to 2024, corresponding to the period in which adjuvant and neoadjuvant therapies became established. Limiting searches to two major databases may have excluded relevant studies indexed elsewhere or published outside this timeframe. Conference searches were restricted to four recent years, and clinical trials were excluded, with conference abstracts combined in the analysis without clear distinction from full-text articles. Evidence from abstracts may be less complete and may include less methodological detail. Quality appraisal was not formally performed, as the substantial heterogeneity in study designs prevented consistent assessment. Readers are encouraged to interpret the results with this context in mind, recognizing that reliability and generalizability may be impacted. Although the scope was intentionally broad across tumor types, the melanoma-specific subset comprised a modest sample, limiting generalizability.

Overall, this review highlights the lack of a unified definition of “cure” across oncology and underscores the methodological and evidentiary challenges that accompany its application in melanoma. Despite growing use of statistical cure models, particularly in the adjuvant and neoadjuvant settings, consistent data standards, mature follow-up, and biologically grounded definitions are still needed to translate modelling outputs into clinically meaningful concepts of cure.

### 4.5 Conclusions

There remains no consensus on how “cure” should be defined or described in the early-stage treatment of melanoma. Perspectives differ across clinical, academic, and patient communities, reflecting the need to balance individual disease characteristics with population-level modeling evidence. Establishing standardized terminology and biologically grounded definitions will be essential to improve communication, strengthen interpretability, and ensure consistent evaluation of therapies with potential curative intent in melanoma.

## Data Availability

The original contributions presented in the study are included in the article/**Supplementary Material**. Further inquiries can be directed to the corresponding author.

## Footnotes

1 The seven tumor types were: melanoma (stage I-III excluding unresectable stage III); triple-negative breast cancer (stage I-III excluding unresectable stage III); non-small cell lung cancer (squamous and non-squamous), including surgically treated early-stage patients with stage IB-IIIA NSCLC (AJCC V7), corresponding to stage II, IIIA, and resectable IIIB (T3-4N2) NSCLC (AJCC V8), and patients with early-stage disease who are ineligible for surgery and who are receiving radiation therapy in stage I or II (T1 to limited T3, N0, M0) NSCLC (AJCC V8); renal cell carcinoma (stage I-IV; T1-T4, N0/M0); head and neck cancer (stages I-IVA); bladder cancer (muscle invasive and non-muscle invasive) (stage I to III, excluding unresectable stage III); and cutaneous squamous cell carcinoma (stage I to III, excluding unresectable stage III).

2 Percentages do not add up to 100% as one publication discussed “cure” in terms of survival/statistical and complete eradication of cancerous cells (Brandao M, Leao J, Gallardo DI, Bourguignon M. Cure rate models for heterogeneous competing causes. Stat Methods Med Res. 2023;32(9):1823–41).

3 Toffolutti 2023 encompassed studies already listed in **Table 1** and was therefore excluded to avoid duplication.

4 This publication (Toffolutti 2023) was not included in **Table 1** because it encompassed other studies already listed in the table, avoiding duplication of information.
