# Supplementary material for: Defining and modeling ‘cure’ in early-stage melanoma: perspectives from a systematic literature review
Source: Front Oncol. 2026 Aug 14;16:1824831. doi: 10.3389/fonc.2026.1824831 (PMC13521801; doi:10.3389/fonc.2026.1824831)
Supplement: Supplementary file 1 [file Supplementaryfile1.docx]

Supplementary Material

# 1 Appendix A: Articles identified for non-melanoma tumor types

**Table 1. Articles identified for non-melanoma tumor types (in addition to melanoma specific)**

| # | Author | Year | Title | Source |
| --- | --- | --- | --- | --- |
| 1 | Rahimzadeh, et al. | 2014 | Estimation of the cure rate in Iranian breast cancer patients | Asian Pacific Journal of Cancer Prevention |
| 2 | Stedman et al | 2014 | Current Estimates of the Cure Fraction: A Feasibility Study of Statistical Cure for Breast and Colorectal Cancer | Journal of the National Cancer Institute Monographs |
| 3 | Ito et al | 2015 | Trends in cure proportion of major cancer sites in Japan between 1993 and 2006 (J-CANSIS study): An observational study | European Journal of Cancer |
| 4 | Tralongo et al. | 2015 | Use of the word “cured” for cancer patients—implications for patients and physicians: the Siracusa charter | Current Oncology |
| 5 | Baghestani, et al. | 2015 | Application of a Non-Mixture Cure Rate Model for Analyzing Survival of Patients with Breast Cancer | Asian Pacific Journal of Cancer Prevention |
| 6 | Beesley, et al. | 2016 | Multiple imputation of missing covariates for the Cox proportional hazards cure model. | Statistics in Medicine |
| 7 | Suh et al | 2017 | Concept analysis on cures for illnesses with high disease burden. | Value Health |
| 8 | Asano, et al. | 2017 | Assessing the prediction accuracy of a cure model for censored survival data with long-term survivors: Application to breast cancer data | Pharmaceutical Statistics |
| 9 | Wu et al | 2017 | Multiple imputation for cure rate quantile regression with censored data | Biometric Journal |
| 10 | Cameron et al | 2018 | Health technology assessment agency appraisal of immuno-oncology cure fraction survival modelling | Value Health |
| 11 | Boussari, et al. | 2018 | A new approach to estimate time-to-cure from cancer registries data | Cancer Epidemiology |
| 12 | Tralongo et al. | 2019 | Major patterns of cancer cure: Clinical implications | European Journal of Cancer Care |
| 13 | Beesley et al. | 2019 | EM algorithms for fitting multistate cure models | Biostatistics |
| 14 | Monnickendam et al | 2020 | A conceptual framework for the approach to the evaluation of oncology survival benefit in health technology assessment | Value Health  (ISPOR 2020 EU) |
| 15 | Shi, et al. | 2020 | Promoting sign consistency in the cure model estimation and selection. | Statistical Methods in Medical Research |
| 16 | Gu et al | 2020 | Semiparametric estimation of the cure fraction in population-based cancer survival analysis | Statistics in Medicine |
| 17 | Morgan et al. | 2021 | What is the Definition of Cure in Non-small Cell Lung Cancer? | Oncology and Therapy |
| 18 | Micallef et al | 2021 | Capturing the Value of Potentially Curative Oncology Therapies: Lessons from the Use and Acceptance of Cure Modelling Assumptions in NICE Technology Appraisals | Value in Health |
| 19 | Pal, et al. | 2021 | A simplified stochastic EM algorithm for cure rate model with negative binomial competing risks: An application to breast cancer data. | Statistics in Medicine |
| 20 | Gandjour et al. | 2021 | The price of curing cancer | BMC Health Services Research |
| 21 | Wang et al | 2022 | A two-way flexible generalized gamma transformation cure rate model | Statistics in Medicine |
| 22 | Safari et al | 2022 | Nonparametric kernel estimation of the probability of cure in a mixture cure model when the cure status is partially observed | Statistical Methods in Medical Research |
| 23 | Hardenberg et al | 2022 | The value of disease free survival (DFS) and osimertinib in adjuvant non-small cell lung cancer (NSCLC): An international Delphi consensus report | Annals of Oncology |
| 24 | Padgett et al | 2022 | Challenges in Economic Modelling of Adjuvant Cancer Therapy for Health Technology Assessments | Value Health  (ISPOR 2020 EU) |
| 25 | Hathway et al | 2022 | Implications of Cure Fractions on Costs of Cancer Care: An Innovative Application to Multi-Cancer Early Detection (MCED) Economic Modeling | Value in Health |
| 26 | Cortes et al | 2022 | The Impact of the Proportion of Cured and Length of Follow-up on Mixture Cure Models (MCMS): A Simulation Study | Value in Health |
| 27 | Collacott et al | 2023 | Patient and clinician perspectives on adjuvant treatment in early-stage non-small cell lung cancer (NSCLC): Qualitative results | Journal of Thoracic Oncology |
| 28 | Plessala et al | 2023 | Cost-effectiveness analysis of atezolizumab as adjuvant treatment of patients with stage II-IIIA non-small cell lung cancer, PD-L1+≥50% of tumor cells in France: A modeling study | Lung Cancer |
| 29 | Omer et al | 2023 | Non-Mixture Cure Model Estimation in Bladder Cancer Patients: A Novel Approach with Exponentiated Weibull Exponential Distribution. | Asian Pacific Journal of Cancer Prevention |
| 30 | Sweeting et al | 2023 | Survival Extrapolation Incorporating General Population Mortality Using Excess Hazard and Cure Models: A Tutorial. | Medical Decision Making |
| 31 | Orozco-Leal et al | 2023 | Considerations for the cure assumption in an NICE single technology appraisal of Nivolumab with chemotherapy for neoadjuvant treatment of resectable Non‑Small Cell Lung Cancer: Evidence assessment group perspective | PharmacoEconomics |
| 32 | Harris et al | 2023 | Cost-effectiveness of nivolumab + platinum doublet chemotherapy as neoadjuvant treatment for resectable non-small cell lung cancer in the United States | Journal of Managed Care & Specialty Pharmacy |
| 33 | Alexopoulos et al | 2023 | A Retrospective Review of Recent NICE Technology Appraisals From 2021 to 2023 to Investigate the Consideration and Acceptability of Cure Modelling Assumptions | Value in Health |
| 34 | Huang et al | 2023 | Cost-Effectiveness of Neoadjuvant Pembrolizumab Plus Chemotherapy Followed by Adjuvant Single-Agent Pembrolizumab for High-Risk Early-Stage Triple-Negative Breast Cancer in the United States | Advances in Therapy |
| 35 | Young et al | 2023 | When Should I Mix? Improving Understanding of the Predictive Behavior of Mixture-Cure Survival Models Informed by Immature Data | Value in Health |
| 36 | Jiang et al | 2024 | Cure models with adaptive activation for modeling cancer survival. | Statistical Methods in Medical Research |
| 37 | Latimer et al | 2024 | Mixture and Non‑mixture Cure Models for Health Technology Assessment: What You Need to Know | PharmacoEconomics |

# 2 Appendix B: PICOTS criteria

Table 2. PICOTS criteria for study inclusion

| **PICOTS element** | **Description** |
| --- | --- |
| Population* | Adults (≥18 years of age) diagnosed with early-stage cancer with any of the following tumor types:   - Melanoma (stage I to III, excluding unresectable stage III) - TNBC (stage I to III, excluding unresectable stage III) - NSCLC (squamous and non-squamous), including:   - Surgically treated early-stage patients with stage IB-IIIA NSCLC (AJCC V7), corresponding to stage II, IIIA, and resectable IIIB (T3-4N2) NSCLC (AJCC V8)   - Patients with early-stage disease who are ineligible for surgery and who are receiving radiation therapy in stage I or II (T1 to limited T3, N0, M0) NSCLC (AJCC V8) - RCC (stage I-IV; T1-T4, N0/M0) - Head and neck cancer (stages I-IVA) - Bladder cancer (muscle invasive and non-muscle invasive) (stage I to III, excluding unresectable stage III) - Cutaneous squamous-cell carcinoma (cSCC) (stage I to III, excluding unresectable stage III) |
| Intervention | Eligibility for primary definitive (curative intent) treatment coupled with neoadjuvant therapies, adjuvant therapies or combinations of those such as neoadjuvant therapy before definitive treatment followed by adjuvant therapy^1^ after definitive treatment.  The focus is on how cure is defined in early-stage settings for patients eligible for systemic intervention before and after primary definitive (curative intent) treatment, but given the limited information, additional insights may be obtained through studies that are based solely on definitive treatments (e.g., interventions like surgery, RT or CRT). Those studies will be identified in the search strategy but will not be included as part of the title/abstract screening process (at least initially). They may be screened at a later phase in case further information is needed. |
| Comparator | No restriction |
| Outcomes | - Explicit and implicit definitions of cure - Clinical criteria considered when identifying cure - Modeling of cure and evidence needs to support assumptions |
| Time frame | 10 years |
| Study design | Including but not limited to: clinical studies, cost-effectiveness analyses, real-world evidence studies, population-based studies, systematic literature reviews. |
| Other | English language, no restriction in geography |

*Note the PICOTS for all tumor indications in the original study have been listed. Abbreviations: AJCC V7: American Joint Committee on Cancer, Version 7 (cancer staging systems); CRT, chemoradiotherapy; cSCC: cutaneous squamous cell carcinoma; NSCLC: non-small cell lung cancer; PICOTS, population, intervention, comparator, outcomes, timeframe, and study design; RCC: renal cell carcinoma; RT, radiation therapy; TNBC: triple-negative breast cancer.

Table 3. PICOTS for study exclusion

| **PICOTS element** | **Potential exclusion criteria** |
| --- | --- |
| Population | - Pediatric-only populations as the focus (or where data on adult patients cannot be identified) - Advanced stage cancer as the focus (or where data on early-stage cancer cannot be identified)   - Metastatic cancer as the focus (or where separate data on non-metastatic cancer cannot be identified) |
| Intervention | Not applicable |
| Comparator | Not applicable |
| Outcomes | Not applicable |
| Time frame | Not applicable |
| Study design | Case reports, in vitro, in vivo studies, letters |

Abbreviations: PICOTS, population, intervention, comparator, outcomes, timeframe, and study design.

# 3 Appendix C: SLR search details

Table 4. SLR search details in PubMed

## 3.1 Supplementary Figures

| **Topic** | **#** | **Number of hits** | **Number of findings** |
| --- | --- | --- | --- |
| Oncology | 1 | ("neoplasms"[MeSH Terms] OR "neoplasms"[All Fields] OR "oncology"[All Fields] OR "oncology s"[All Fields] OR "cancer*"[All Fields] OR "solid tumor*"[All Fields] OR "solid tumour*"[All Fields] OR "malign"[All Fields] OR "malignance"[All Fields] OR "malignances"[All Fields] OR "malignant"[All Fields] OR "malignants"[All Fields] OR "malignities"[All Fields] OR "malignity"[All Fields] OR "malignization"[All Fields] OR "malignized"[All Fields] OR "maligns"[All Fields] OR "neoplasms"[MeSH Terms] OR "neoplasms"[All Fields] OR "malignancies"[All Fields] OR "malignancy"[All Fields] OR "carcinoma"[MeSH Terms] OR "carcinoma"[All Fields] OR "carcinomas"[All Fields] OR "carcinoma s"[All Fields]) | 5,477,631 |
| Type of cancer & disease stage | 2 | ("melanoma"[MeSH Terms] OR "melanoma"[All Fields] OR "melanomas"[All Fields] OR "melanoma s"[All Fields] OR ("skin"[MeSH Terms] OR "skin"[All Fields]) OR ("lung"[MeSH Terms] OR "lung"[All Fields]) OR ("carcinoma, non small cell lung"[MeSH Terms] OR ("carcinoma"[All Fields] AND "non small cell"[All Fields] AND "lung"[All Fields]) OR "non-small-cell lung carcinoma"[All Fields] OR "nsclc"[All Fields] OR "nsclc s"[All Fields] OR "nsclcs"[All Fields]) OR "renal cell carcinoma"[All Fields] OR "RCC"[All Fields] OR ("breast"[MeSH Terms] OR "breast"[All Fields] OR "breasts"[All Fields] OR "breast s"[All Fields]) OR "TNBC"[All Fields] OR "triple*negative breast cancer"[All Fields] OR ("bladder s"[All Fields] OR "urinary bladder"[MeSH Terms] OR ("urinary"[All Fields] AND "bladder"[All Fields]) OR "urinary bladder"[All Fields] OR "bladder"[All Fields] OR "bladders"[All Fields]) OR "head and neck"[All Fields] OR "head and neck squamous cell carcinoma"[All Fields] OR ("hnsccs"[All Fields] OR "squamous cell carcinoma of head and neck"[MeSH Terms] OR ("squamous"[All Fields] AND "cell"[All Fields] AND "carcinoma"[All Fields] AND "head"[All Fields] AND "neck"[All Fields]) OR "squamous cell carcinoma of head and neck"[All Fields] OR "hnscc"[All Fields]) OR ("scchn"[All Fields] OR "scchns"[All Fields]) OR "cSCC"[All Fields] OR "cutaneous squamous cell carcinoma"[All Fields] OR (("cutaneous"[All Fields] OR "cutaneously"[All Fields] OR "cutanous"[All Fields]) AND ("carcinoma, squamous cell"[MeSH Terms] OR ("carcinoma"[All Fields] AND "squamous"[All Fields] AND "cell"[All Fields]) OR "squamous cell carcinoma"[All Fields] OR ("squamous"[All Fields] AND "cell"[All Fields] AND "carcinoma"[All Fields]))) OR ("neoadjuvancy"[All Fields] OR "neoadjuvant therapy"[MeSH Terms] OR ("neoadjuvant"[All Fields] AND "therapy"[All Fields]) OR "neoadjuvant therapy"[All Fields] OR "neoadjuvant"[All Fields] OR "neoadjuvants"[All Fields] OR "neoadjuvent"[All Fields]) OR "Neo-adjuvant"[All Fields] OR ("adjuvancy"[All Fields] OR "adjuvanted"[All Fields] OR "adjuvanting"[All Fields] OR "adjuvants"[All Fields] OR "adjuvants pharmaceutic"[Pharmacological Action] OR "adjuvants immunologic"[Pharmacological Action] OR "adjuvants, pharmaceutic"[MeSH Terms] OR ("adjuvants"[All Fields] AND "pharmaceutic"[All Fields]) OR "pharmaceutic adjuvants"[All Fields] OR "adjuvant"[All Fields] OR "adjuvants, immunologic"[MeSH Terms] OR ("adjuvants"[All Fields] AND "immunologic"[All Fields]) OR "immunologic adjuvants"[All Fields] OR "adjuvated"[All Fields] OR "adjuvation"[All Fields] OR "adjuvent"[All Fields]) OR "resect*"[All Fields] OR "early"[All Fields]) | 5,522,556 |
|  | 3 | 1 AND 2 | 2,111,715 |
| Definition | 4 | (("guideline"[Publication Type] OR "guidelines as topic"[MeSH Terms] OR "guideline*"[All Fields]) OR ("consensual"[All Fields] OR "consensually"[All Fields] OR "consensus"[MeSH Terms] OR "consensus"[All Fields]) OR "delphi"[All Fields] OR ("definite"[All Fields] OR "definitely"[All Fields] OR "definition"[All Fields] OR "definitional"[All Fields] OR "definitions"[All Fields] OR "definitive"[All Fields] OR "definitively"[All Fields]) OR "clinical criteria"[All Fields] OR ("review"[Publication Type] OR "review literature as topic"[MeSH Terms] OR "review"[All Fields]) OR "perspective*"[All Fields] OR "concept*"[All Fields] OR ("approach"[All Fields] OR "approach s"[All Fields] OR "approachability"[All Fields] OR "approachable"[All Fields] OR "approache"[All Fields] OR "approached"[All Fields] OR "approaches"[All Fields] OR "approaching"[All Fields] OR "approachs"[All Fields]) OR ("concept formation"[MeSH Terms] OR ("concept"[All Fields] AND "formation"[All Fields]) OR "concept formation"[All Fields] OR "conceptualisation"[All Fields] OR "conceptualization"[All Fields] OR "conceptual"[All Fields] OR "conceptualisations"[All Fields] OR "conceptualise"[All Fields] OR "conceptualised"[All Fields] OR "conceptualises"[All Fields] OR "conceptualising"[All Fields] OR "conceptualism"[All Fields] OR "conceptualizations"[All Fields] OR "conceptualize"[All Fields] OR "conceptualized"[All Fields] OR "conceptualizes"[All Fields] OR "conceptualizing"[All Fields] OR "conceptually"[All Fields]) OR ("hypothesis tor"[Journal] OR "hypothesis macon"[Journal] OR "hypothesis"[All Fields]) OR ("idea j law technol"[Journal] OR "idea"[All Fields]) OR ("notion"[All Fields] OR "notion s"[All Fields] OR "notions"[All Fields]) OR ("terminologies"[All Fields] OR "terminology"[All Fields]) OR ("interpret"[All Fields] OR "interpretability"[All Fields] OR "interpretable"[All Fields] OR "interpretating"[All Fields] OR "interpretation"[All Fields] OR "interpretation s"[All Fields] OR "interpretational"[All Fields] OR "interpretations"[All Fields] OR "interpretative"[All Fields] OR "interpreted"[All Fields] OR "interpreter"[All Fields] OR "interpreter s"[All Fields] OR "interpreters"[All Fields] OR "interpreting"[All Fields] OR "interpretive"[All Fields] OR "interpretively"[All Fields] OR "interprets"[All Fields]) OR ("rationale"[All Fields] OR "rationales"[All Fields]) OR "treatment goal"[All Fields]) | 8,777,507 |
| Cure | 5 | ("cure*"[All Fields] OR "cure rate*"[All Fields] OR "statistical cure"[All Fields] OR "cure model*"[All Fields] OR "cure modelling"[All Fields] OR "cure fraction*"[All Fields] OR "cure potential"[All Fields] OR "curative intent"[All Fields] OR "curative"[All Fields] OR "curatively"[All Fields] OR "curativity"[All Fields] OR "long*term cure"[All Fields] OR "functional cure"[All Fields] OR "long-term survival"[All Fields] OR "long-term survival"[All Fields] OR "remission"[All Fields] OR "remissions"[All Fields] OR "continuous remission"[All Fields] OR "sustained remission"[All Fields] OR "lasting response"[All Fields] OR "durable response"[All Fields] OR "sustained response"[All Fields] OR "late recurrence"[All Fields] OR "ultra-late recurrence"[All Fields] OR "disease specific mortality"[All Fields] OR "time-to-cure"[All Fields] OR "time-to-cure"[All Fields]) | 572,354 |
|  | 6 | 4 AND 5 | 198,604 |
|  | 7 | 3 AND 6 | 50,748 |
|  | 8 | 7 applying the following filter: English, from 2014 - 2024 | 24,542 |
|  |  |  |  |

Table 5. SLR search details in EMBASE

| **Topic** | **#** | **Terms** | **Number of findings** |
| --- | --- | --- | --- |
| Oncology | 1 | 'oncology' OR 'oncology'/exp OR cancer* OR 'solid tumor*' OR 'solid tumour*' OR 'malignancy'/exp OR malignancy OR carcinoma | 6,431,248 |
| Type of cancer & disease stage | 2 | 'melanoma'/exp OR 'melanoma' OR ‘skin’/exp OR ‘skin’ OR 'lung'/exp OR 'lung' OR 'nsclc' OR 'renal cell carcinoma'/exp OR 'renal cell carcinoma' OR 'rcc' OR 'breast'/exp OR ‘breast’ OR 'tnbc'/exp OR ‘tnbc’ OR ‘triple*negative breast cancer’ OR 'bladder'/exp OR ‘bladder’ OR 'head and neck' OR 'head and neck squamous cell carcinoma'/exp OR 'head and neck squamous cell carcinoma' OR 'hnscc'/exp OR ‘hnscc’ OR 'scchn' OR ‘cutaneous squamous cell carcinoma’/exp OR ‘cutaneous squamous cell carcinoma’ OR cSCC OR 'neoadjuvant' OR 'neo-adjuvant' OR 'adjuvant'/exp OR 'adjuvant' OR 'resectable' OR 'early' | 8,009,031 |
|  | 3 | 1 AND 2 | 2,770,231 |
| Definition | 4 | 'guideline*' OR 'consensus'/exp OR 'consensus' OR 'delphi'/exp OR 'delphi' OR 'definition'/exp OR 'definition' OR 'clinical criteria'/exp OR 'clinical criteria' OR 'review'/exp OR 'review' OR 'perspective*' OR 'concept*' OR ‘approach’ OR ’conceptual’ OR ‘hypothesis’/exp OR hypothesis OR idea OR notion OR ‘terminology’/exp OR terminology OR ‘interpretation’/exp OR ‘interpretation’ OR rationale OR 'treatment goal' | 11,085,472 |
| Cure | 5 | 'cure*' OR 'cure rate*' OR 'statistical cure' OR 'cure model*' OR 'cure modelling' OR 'cure modeling' OR 'cure fraction*' OR 'cure potential' OR 'curative intent' OR 'curative' OR 'long*term cure' OR 'functional cure' OR 'long*term survival' OR 'remission'/exp OR ‘remission’ OR 'continuous remission' OR 'sustained remission'/exp OR ‘sustained remission’ OR 'lasting response' OR 'durable response' OR 'sustained response'/exp OR ‘sustained response’ OR ‘late recurrence’/exp OR ‘late recurrence’ OR ‘ultra late recurrence’ OR ‘disease specific mortality’/exp OR ‘disease specific mortality’ OR ‘time to cure’ OR ‘time-to-cure’ | 791,112 |
|  | 6 | 4 AND 5 | 265,153 |
|  | 7 | 3 AND 6 | 60,292 |
|  | 8 | 7 applying the following filter: English, from 2014 - 2024 | 37,341 |
|  |  |  |  |

Table 6. Searches for conference abstract database/documents

| **Topic** | **Key words** |
| --- | --- |
| Oncology | Cancer, oncology, malignancy |
| Type of cancer & disease stage | Neoadjuvant, adjuvant |
| Definition | Not applicable |
| Cure | Cure, curative |

Table 7.Total number of hits per conference

| Conference | Number of findings | Melanoma specific |
| --- | --- | --- |
| ISPOR | 7 | 4 |
| ESMO | 1 | 0 |
| ASCO | 0 | 0 |

Abbreviations: ASCO, American Society of Clinical Oncology; ESMO, European Society for Medical Oncology; ISPOR, International Society for Pharmacoeconomics and Outcomes Research.

# 4 Appendix D: Detailed Description of AI-Assisted Screening and Quality Assurance Procedures

Training details

At Level 1, title and abstract screening were conducted making use of an artificial intelligence (AI) -assisted approach (i.e., human reviewer + AI) on the web-based systematic review platform. First, two reviewers conducted screening of a sample of 50 articles, the minimum sample size needed to train the AI screening model. They independently screened each of the articles and any discrepancies were discussed and resolved ensuring alignment to minimize discrepancies in the inclusion and exclusion decisions. The AI-based screening model was then trained on these 50 articles. The requirement for 50 articles, including 10 inclusions, ensures the AI model learned from a diverse set of examples. This diversity helped the AI recognize the nuances of inclusion and exclusion criteria, improving accuracy in identifying relevant articles.

Model calibration metrics

Based on the final decision made on these articles (i.e., include/exclude), prediction AI then anticipated which of the remaining articles were most relevant to the research question. Human involvement was crucial to ensure success of AI alignment algorithms. Fifteen more articles were screened to optimize the program where the area under the curve (AUC) of ≥0.98 was achieved (indicating that the AI model is effectively discerning between abstracts which should be advanced to full text screening and those which should be excluded). An AUC of ≥0.8 is recommended to achieve a robust model performance, so only the studies that contain information relevant to the research question are passed to the next phase of screening. The model was then run on the remaining title/abstracts to predict their further inclusion in the study.

Inclusion threshold

An appropriate threshold of 0.25 for inclusion was identified and all the articles predicted for inclusion above this threshold were then screened by a human reviewer to determine final inclusion/exclusion. Only those passing both AI and human screening were passed to level 2 screening. A sample of 100 excluded articles which were closest to the AI inclusion threshold were also reviewed by a human to confirm their exclusion for quality assurance purposes.

Full-text screening details

At Level 2, following the title and abstract screening, a full-text screening was conducted by two independent (human) reviewers according to the pre-defined inclusion criteria and exclusion criteria). A third independent reviewer was consulted in case of disagreement. Articles had to meet all the inclusion criteria and none of the exclusion criteria to be included. As with Level 1 screening, reasons for exclusion of studies were captured for each article.
